# Variation in the Respiration-to-Photosynthesis Ratio of Chinese Fir Saplings Is Associated with Foliar Non-Structural Carbohydrates

**DOI:** 10.3390/plants15121800

**Published:** 2026-06-11

**Authors:** Liang Fang, Zhenning Ding, Siyu Zhu, Wenjun Hu, Haoxiang Tang, Yingjie Chen, Qingyong Lin, Kai Wang, Shubin Li, Yanghui Fang

**Affiliations:** 1 College of Forestry, Fujian Agriculture and Forestry University, Fuzhou 350002, China; 2 Fujian Provincial Forestry Science and Technology Promotion General Station, Fuzhou 350003, China

**Keywords:** carbon balance, carbon budgets, *Cunninghamia lanceolata*, plantation forest, photosynthesis, respiration, soluble sugars, starch

## Abstract

Foliar respiration-to-photosynthesis ratio (R/A) is a key indicator of carbon balance and has been widely used to infer plant productivity and carbon sequestration potential. Yet, the extent to which R/A varies among genotypes within a species remains unclear. Chinese fir (*Cunninghamia lanceolata* (Lamb.) Hook.) is a dominant plantation conifer species in southern China. Here, we assessed genotypic variation in R/A and investigated its potential drivers in saplings of three Chinese fir genotypes (Gen3, Y020 and Y061) grown under uniform greenhouse conditions. R/A varied among genotypes, and the variation was driven mainly by differences in respiration, although photosynthesis also varied among genotypes. Compared to Gen3 and Y061, Y020 showed the highest R/A, indicating greater respiratory carbon loss relative to carbon gain. Across genotypes, respiration was positively associated with foliar nitrogen concentration (foliar N) and non-structural carbohydrate concentrations (NSC; including soluble sugars and starch), whereas photosynthesis was unrelated to foliar N and NSC. More importantly, R/A increased with NSC but not with foliar N. Overall, these results reveal substantial genotypic variation in R/A in Chinese fir saplings and suggest that NSC is an important factor associated with foliar carbon balance. Our findings have practical implications for selecting genotypes to enhance carbon sink capacity and stress resilience in plantation management.

## 1. Introduction

Forest ecosystems are central to the global carbon cycle, serving as major terrestrial carbon sinks and playing a critical role in climate regulation [1,2]. Plantation forests, in particular, have attracted growing attention because of their high productivity and substantial potential for large-scale carbon storage [3,4]. Chinese fir (*Cunninghamia lanceolata* (Lamb.) Hook.), a fast-growing evergreen coniferous tree species, is the most widely planted tree in southern China, covering more than 11 million hectares [5,6,7,8]. Chinese fir is essential not only for timber production but also for ecological restoration and carbon sequestration. Therefore, as climate change accelerates and the demand for ecosystem services increases, enhancing the productivity and carbon sink capacity of Chinese fir plantations through scientific management and improved breeding will be vital for reconciling economic development with ecological sustainability [9,10].

Leaves are the primary organs where both photosynthesis and respiration occur [11,12]. The respiration-to-photosynthesis ratio (R/A) is an important physiological trait that reflects carbon balance at the leaf scale. This ratio, analogous to carbon use efficiency at the stand or ecosystem scale, provides valuable insights into the balance between carbon gain through photosynthesis and carbon loss through respiration, and serves as a key indicator for assessing vegetation productivity [13,14,15,16,17]. In fact, a substantial proportion of the carbon fixed by photosynthesis, ranging from 21% to 78%, is ultimately respired back to the atmosphere by plants [18,19,20]. Nonetheless, it has long been assumed that the proportion of respiration to photosynthesis is conservative [21,22,23,24]. However, with the accumulation of experimental data and observations, mounting recent evidence suggests that, although relatively conservative, R/A exhibits substantial variability, which may result in uncertainties in ecosystem carbon cycling and productivity assessment [19,25].

For both photosynthesis and respiration, nitrogen (N) is a key nutrient because of its role in the synthesis of essential proteins and enzymes [11,26,27]. While the importance of foliar N for plant carbon metabolism is well recognized, experimental evidence has shown that not only the absolute amount but also the partitioning of N between chloroplasts and mitochondria, can impact plant carbon balance. Specifically, with increasing N supply, a greater proportion of N is allocated to chloroplasts and less to mitochondria, which directly affects R/A and ultimately shapes plant carbon balance [28]. Moreover, non-structural carbohydrates (NSC), mainly including soluble sugars and starch, serve as important carbon pools and can also play crucial roles in regulating foliar carbon balance [19,29]. NSC storage is widely recognized as a reservoir that supplies carbon to sustain respiratory processes in plants [29]. Nonetheless, the regulatory roles of starch and soluble sugars in respiration may differ, since sugars produced during the daytime are stored as starch in chloroplasts when carbon supply exceeds immediate demand, and this stored starch is then mobilized during the nighttime to sustain metabolic activities such as respiration [29,30,31]. In parallel, NSC may also play a role in regulating photosynthesis, as excessive accumulation of NSC may suppress photosynthesis via feedback inhibition, likely due to limited carbohydrate sink capacity [32,33,34,35]. Consequently, studies investigating how foliar nitrogen and NSC influence foliar carbon balance, as well as their specific mechanisms of action, remain relatively limited.

Genotype selection forms an important component of plantation management. In the context of genotype screening and selection, many current studies have focused solely on genetic variation in photosynthetic capacity [36,37,38], while integrated assessments of both carbon acquisition and carbon loss across genotypes remain limited. Focusing solely on photosynthesis would lead to incomplete or biased evaluations of plant carbon budgets [20,39]. Moreover, despite growing attention to plant carbon balance, most current research has primarily focused on the effects of environmental change [15,21,40], with relatively limited attention paid to differences among species or genotypes. Given the central role of Chinese fir in afforestation in southern China, elucidating the physiological basis of its carbon balance is of significant scientific and practical importance [41,42,43].

In this study, we compared three common but contrasting Chinese fir genotypes from Fujian Province in terms of their foliar photosynthesis, respiration, R/A, foliar NSC, as well as foliar carbon (C) and nitrogen (N) concentrations. We hypothesized that: (i) R/A varies among Chinese fir genotypes, and (ii) the variation is associated with foliar N and NSC.

## 2. Materials and Methods

### 2.1. Experimental Location and Plant Materials

The experiment was conducted in the greenhouse of Fujian Agricultural and Forestry University in Fuzhou City, Fujian Province, China (119°24′ E, 26°08′ N). The greenhouse is located in southeastern China, near the East China Sea, and has a humid subtropical climate with distinct seasons.

The experimental materials included three genotypes of Chinese fir (*Cunninghamia lanceolata* (Lamb.) Hook.): Gen3, Y020, and Y061, all from the Yangkou Forest Farm, Nanping City, Fujian Province, China. Gen3 is an elite genotype selected from the third-generation seed orchard at Yangkou Forest Farm through successive cycles of genetic improvement and is characterized by strong growth stability and high stress tolerance. Y020 and Y061 are also elite genotypes developed by the Yangkou Forest Farm. Y020 is characterized by early rapid growth, strong natural pruning ability, and well-formed lateral branches, while Y061 is characterized by rapid growth and high productivity, with consistent and stable diameter and height growth after planting and high timber yield.

### 2.2. Experimental Setup

In March 2025, saplings of three Chinese fir genotypes, propagated by cuttings, were transplanted into pots with a diameter of 30 cm and a height of 23.5 cm. One Chinese fir sapling was planted in each pot. A total of 40 pots were established, including 20 pots of Gen3, 12 pots of Y020, and 8 pots of Y061. All saplings were cultivated under natural conditions in a greenhouse, with appropriate watering and fertilization to ensure normal growth. Ultimately, 17 healthy saplings with uniform growth (i.e., plant height and basal diameter were similar within each genotype) were selected for the experiment, consisting of 6 pots of Gen3, 5 pots of Y020, and 6 pots of Y061. The remaining saplings were randomly distributed among all experimental pots to minimize confounding effects.

Measurements and sampling were conducted between October 27th and 29th, during the second growth peak period of Chinese fir (August to November) [44]. From October 24th to 29th, a HOBO data logger (MX1104, Onset Inc., Bourne, MA, USA) was installed in the greenhouse to record microclimatic conditions at hourly intervals. During this period, the average temperature and relative humidity were 24.7 °C and 68.31%, respectively.

### 2.3. Photosynthetic and Respiration Rates

A portable photosynthetic system (Li-Cor 6800; Li-Cor Inc., Lincoln, NE, USA) equipped with a 6 × 6 cm^2^ needle chamber was used to measure foliar photosynthetic and respiration rates. Measurements were performed between 8:30 and 12:00 h. The measurement order was randomly adjusted to minimize time and sequence effects. For light-saturated photosynthetic rate, the environmental parameters in the cuvette were set as follows: photosynthetically active radiation (PAR) at 1500 μmol m^−2^ s^−1^, reference CO_2_ concentration at 400 ppm, chamber temperature at 25 °C, and relative humidity at 60%, resulting in a leaf-to-air vapor pressure deficit of approximately 1.5 kPa. After photosynthetic rate measurements, dark respiration was immediately measured on the same needle by switching off the light in the cuvette. The environmental parameters in the cuvette were identical to those used for photosynthetic rate measurements, except that PAR was set to 0 μmol m^−2^ s^−1^. For both photosynthesis and respiration measurements, data were recorded after all gas exchange parameters reached a steady state (for a minimum of 30 min for photosynthesis and a minimum of 10 min for respiration).

Because the measured Chinese fir needles did not fully cover the chamber, a post-measurement leaf area correction was applied [45]. After gas exchange measurements, foliar samples were collected, and the projected leaf area of the measured needles was determined using an Epson V19 scanner (Epson Perfection V19, Epson Inc., USA) and WinRHIZO version 2022a (Regent Instruments Inc., Canada). Photosynthetic and respiration rates were subsequently corrected for leaf area before analyses. The remaining foliar samples were used to determine foliar dry mass, as well as for analyses of foliar carbon and nitrogen concentrations, and foliar soluble sugars and starch concentrations (see below sections).

### 2.4. Calculation of Mass-Based Gas Exchange Rates

The needle material used for gas exchange and leaf area measurements was oven-dried at 75 °C to a constant weight and subsequently weighed. Specific leaf area (SLA, cm^2^ g^−1^) was calculated as SLA = LA/LDW, where LA (cm^2^) is the leaf area and LDW (g) is the leaf dry weight.

Based on evidence that mass-based expressions of foliar nutrient concentrations and photosynthetic and respiration rates are more closely correlated with relative plant growth rates than area-based expressions [46,47,48], all measured photosynthetic and respiration rates (umol m^−2^ s^−1^) on an area basis were converted to mass-based rates (nmol g^−1^ s^−1^) using SLA data, yielding mass-based photosynthetic (Am, nmol g^−1^ s^−1^) and respiration (Rm, nmol g^−1^ s^−1^) rates.

### 2.5. Foliar Carbon and Nitrogen Concentrations

The concentrations of total carbon (foliar C) and nitrogen (foliar N) in needle material on a mass basis (mg g^−1^) were determined simultaneously using an EA1108 CHN-O Elemental Analyzer (Vario Max, Elementar, Germany) based on the Dumas combustion method.

### 2.6. Foliar Soluble Sugars and Starch Concentrations 

The concentrations of soluble sugars and starch (mg g^−1^) were determined using the anthrone colorimetric method. Soluble sugars were extracted from the samples with 80% ethanol to separate them from starch.

### 2.7. Statistical Analysis

All data were tested for normality and homogeneity of variance prior to analysis. One-way ANOVA was used to examine differences in parameters/traits among genotypes. When significant effects were detected, pairwise comparisons between genotypes were conducted using Tukey’s HSD test. Linear regression analysis was conducted to assess the relationships between foliar NSC and nitrogen and carbon fluxes. Principal component analysis (PCA) was used to integrate leaf physiological and biochemical parameters/traits, visualizing clustering patterns among genotypes. The significance level was set at *p* < 0.05. All statistical analyses were performed using R version 4.5.0 (R Foundation).

## 3. Results

### 3.1. Mass-Based Foliar Photosynthesis, Respiration, and Their Ratio

**Figure 1:**
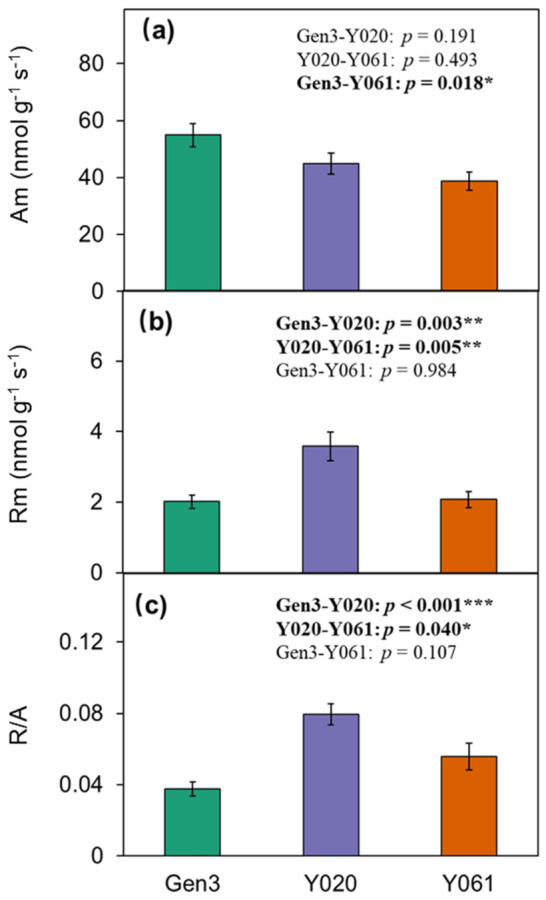
Mass-based foliar photosynthetic and respiration rates and their ratio in three Chinese fir genotypes Gen3, Y020, and Y061. (**a**) Mass-based photosynthetic rate (Am), (**b**) mass-based respiration rate (Rm), (**c**) the respiration-to-photosynthesis ratio (R/A). Values are means ± SE (*n* = 6 for Gen3 and Y061, *n* = 5 for Y020). Statistical differences among genotypes were assessed using one-way ANOVA (Table 1) followed by Tukey’s HSD test. Bold font indicates statistical significance. *, **, and *** indicate significance levels of *p* < 0.05, *p* < 0.01, and *p* < 0.001, respectively.

Significant differences in Am, Rm, and R/A were observed among the three Chinese fir genotypes (Figure 1).

One-way ANOVA analysis revealed a significant genotypic effect on Am (F = 5.06, *p* = 0.022; Table 1). Post hoc comparisons showed that Y061 had a lower Am than Gen3 (*p* = 0.018), while the Am of Y020 did not significantly differ from those of the other two genotypes (Figure 1a). For Rm, genotypic differences were even more pronounced (F = 10.06, *p* = 0.002; Table 1). Both Gen3 and Y061 showed lower Rm compared to Y020 (*p* < 0.01 for both comparisons), whereas no significant difference in Rm was found between Gen3 and Y061 (Figure 1b). R/A also differed among genotypes (F = 11.64, *p* = 0.001; Table 1). Both Gen3 and Y061 had lower R/A compared to Y020 (*p* < 0.04; Figure 1). Although the R/A for Y061 was slightly higher than that for Gen3, the difference was not statistically significant (*p* = 0.109; Figure 1c).

### 3.2. Specific Leaf Area

**Figure 2:**
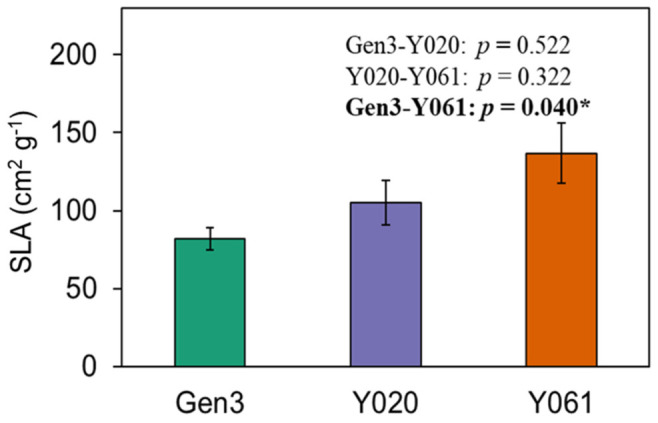
Specific leaf area (SLA) of the three Chinese fir genotypes Gen3, Y020, and Y061. Values are means ± SE (*n* = 6 for Gen3 and Y061, *n* = 5 for Y020). Statistical differences among genotypes were assessed using one-way ANOVA (Table 1) followed by Tukey’s HSD test. Bold font indicates statistical significance. An asterisk (*) indicates statistical significance at *p* < 0.05.

SLA showed genotypic variation (*p* = 0.049; Table 1). Among the three genotypes, SLA tended to be higher in Y061 and lower in Gen3, whereas Y020 displayed intermediate values (Figure 2). Post hoc comparisons revealed that the SLA for Y061 was higher than that for Gen3 (*p* = 0.040), whereas the differences in SLA between Y020 and the other two genotypes were not statistically significant.

### 3.3. Foliar Non-Structural Carbohydrate Concentrations 

**Figure 3:**
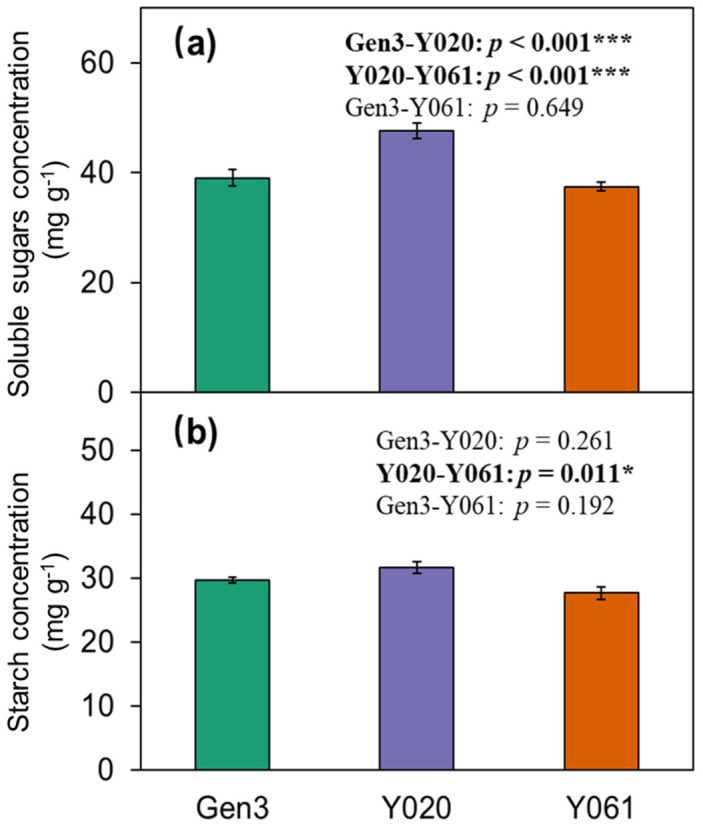
Foliar non-structural carbohydrates in three Chinese fir genotypes Gen3, Y020, and Y061. (**a**) Soluble sugars concentration, (**b**) starch concentration. Values are means ± SE (*n* = 6 for Gen3 and Y061, *n* = 5 for Y020). Statistical differences among genotypes were assessed using one-way ANOVA (Table 1) followed by Tukey’s HSD test. Bold font indicates statistical significance. * and *** indicate significance levels of *p* < 0.05 and *p* < 0.001, respectively.

ANOVA revealed a strong genotypic effect on foliar soluble sugars concentration (*p* <  0.001; Table 1). Post hoc comparisons showed that Y020 had a higher soluble sugars concentration than both Gen3 and Y061; however, no significant difference in foliar soluble sugars concentration was observed between Gen3 and Y061 (Figure 3a).

A comparable pattern was observed for starch concentration (Figure 3b), although compared to soluble sugars concentration, starch concentration showed less genotypic variation (Table 1). The starch concentration differed across genotypes (*p* = 0.013; Table 1), with Y061 having a lower starch concentration than Y020 (*p* = 0.010; Figure 3b). Gen3 showed an intermediate starch concentration but did not differ significantly from Y020 or Y061.

### 3.4. Foliar Carbon, Nitrogen, and Their Derived Ratios

**Figure 4:**
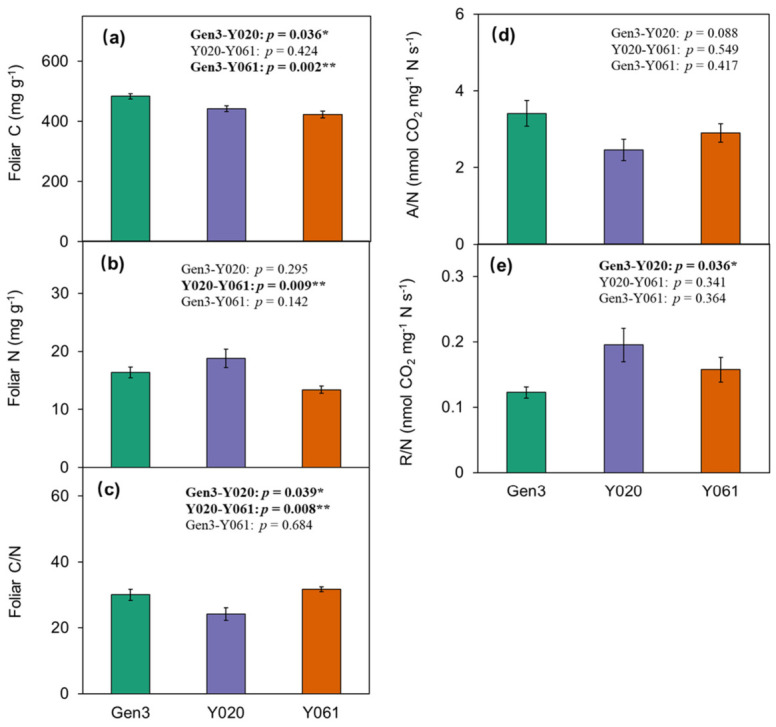
Foliar nutrient concentrations and ratios for the three Chinese fir genotypes Gen3, Y020, and Y061. (**a**) Foliar carbon concentration (foliar C), (**b**) foliar nitrogen concentration (foliar N), (**c**) the ratio of foliar carbon concentration to foliar nitrogen concentration (foliar C/N), (**d**) the ratio of photosynthesis to foliar nitrogen concentration (A/N), and (**e**) the ratio of respiration to foliar nitrogen concentration (R/N). Values are means ± SE (*n* = 6 for Gen3 and Y061, *n* = 5 for Y020). Statistical differences among genotypes were assessed using one-way ANOVA (Table 1) followed by Tukey’s HSD test. Bold font indicates statistical significance. * and ** indicate significance levels of *p* < 0.05 and *p* < 0.001, respectively.

Foliar C differed among genotypes (*p* = 0.002; Table 1). Gen3 had a higher foliar C than Y020 and Y061, with significant pairwise differences observed between Gen3 and both Y020 (*p* = 0.036) and Y061 (*p* = 0.002) (Figure 4a).

Foliar N also varied substantially across genotypes (*p* = 0.012; Table 1), with higher foliar N in Y020 compared to Y061 (*p* = 0.009; Figure 4b). The foliar N in Gen3 was slightly lower than that in Y020, although the difference was not statistically significant (Figure 4b).

A genotypic difference was detected in the ratio of foliar C to foliar N (foliar C/N) (*p* = 0.009; Table 1). Both Gen3 and Y061 showed higher foliar C/N than Y020 (*p* = 0.039 and *p* = 0.008, respectively), whereas the difference in foliar C/N between Gen3 and Y061 was not significant (Figure 4c).

The ratio of Am to foliar N (A/N) followed a pattern similar to that of foliar C/N but did not differ significantly among genotypes (*p* = 0.103; Table 1). Gen3 had the highest A/N, while the A/N in Y020 tended to be lower than that of both Gen3 and Y061 (Figure 4d). For the ratio of Rm to foliar N (R/N), a genotypic effect was detected (*p* = 0.045; Table 1). In contrast to the patterns observed for A/N and C/N, Y020 showed a higher R/N than both Gen3 and Y061 (Figure 4e), although only the difference between Y020 and Gen3 was statistically significant (*p* = 0.036).

### 3.5. Associations of Gas Exchange and Biochemical Traits

**Figure 5:**
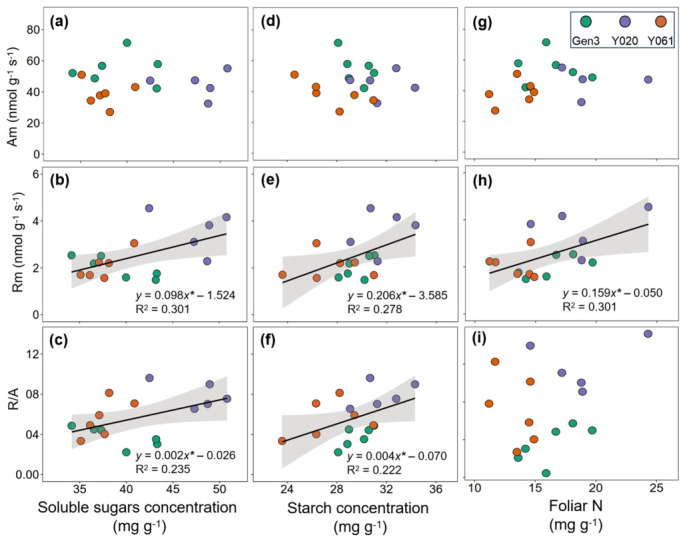
Relationships of mass-based photosynthetic rate (Am), mass-based respiration rate (Rm), and their ratio (R/A) with foliar soluble sugars, starch, and foliar nitrogen (N) concentrations, across three Chinese fir genotypes Gen3, Y020 and Y061. (**a**) the relationship between Am and soluble sugars concentration; (**b**) the relationship between Rm and soluble sugars concentration; (**c**) the relationship between R/A and soluble sugars concentration; (**d**) the relationship between Am and starch concentration; (**e**) the relationship between Rm and starch concentration; (**f**) the relationship between R/A and starch concentration; (**g**) the relationship between Am and foliar N; (**h**) the relationship between Rm and foliar N; and (**i**) the relationship between R/A and foliar N. The shaded areas around the regression lines are 95% confidence intervals of the predictions. An asterisk (*) indicates statistical significance at *p* < 0.05.

Linear regressions were performed to explore potential relationships between leaf carbon gas exchange (Am, Rm and R/A) and either foliar NSC pools (soluble sugars and starch concentrations) or foliar N (Figure 5). No significant relationship was found between soluble sugars concentration and Am (Figure 5a), nor between starch concentration and Am (Figure 5d). By contrast, Rm was positively correlated with both soluble sugars (*p* = 0.023) and starch (*p* = 0.030) concentrations (Figure 5b,e). Similarly, R/A was positively associated with soluble sugars (*p* = 0.049) and starch (*p* = 0.050) concentrations (Figure 5c,f). Regarding foliar N, no significant relationship was observed between Am and foliar N (*p* = 0.352) (Figure 5g). In contrast, Rm showed a positive relationship with foliar N (*p* = 0.023) (Figure 5h). There was no significant relationship between R/A and foliar N (*p* = 0.225) (Figure 5i).

**Figure 6:**
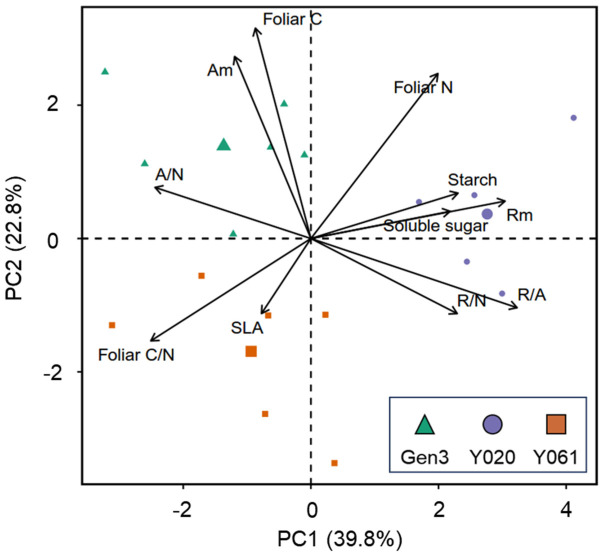
Principal component analysis of foliar gas exchange and physiological traits for three Chinese fir genotypes Gen3, Y020 and Y061. Abbreviations and units: Am, mass-based photosynthetic rate (nmol g^−1^ s^−1^); Rm, mass-based respiration rate (nmol g^−1^ s^−1^); R/A, the respiration-to-photosynthesis ratio (unitless); SLA, specific leaf area (cm^2^ g^−1^); Foliar N, foliar nitrogen concentration (mg g^−1^); Foliar C, foliar carbon concentration (mg g^−1^); Foliar C/N, the ratio of foliar carbon concentration to foliar nitrogen concentration (unitless); A/N, the ratio of photosynthesis to foliar nitrogen concentration (nmol CO_2_ mg^−1^ N s^−1^); R/N, the ratio of respiration to foliar nitrogen concentration (nmol CO_2_ mg^−1^ N s^−1^). The contribution (%) of each PCA axis (PC1 and PC2) is indicated on the figure.

PCA was conducted based on the studied traits across all genotypes. The first two principal components (PC1 and PC2) together explained 62.6% of the total variance, accounting for 39.8% and 22.8%, respectively (Figure 6). PC1 was mainly characterized by positive loadings from Rm, R/A, R/N, foliar N, soluble sugars and starch concentrations, while A/N and foliar C/N showed negative contributions. PC2 was positively associated with Am, foliar C and foliar N, but negatively with SLA and foliar C/N (Figure 6).

PCA also revealed clear clustering patterns among genotypes. Along PC1, Y020 individuals were clearly separated in the positive direction, aligning with vectors for Rm, R/A, R/N, soluble sugars and starch concentrations, and foliar N (Figure 6). In contrast, Gen3 and Y061 clustered in the negative direction, along vectors for A/N and foliar C/N. Along PC2, Gen3 individuals were located in the upper part of the plot, associated with Am, foliar C and foliar N, whereas Y061 individuals were located in the lower part, and Y020 individuals remained near the origin (Figure 6).

## 4. Discussion

### 4.1. Variation in Foliar Carbon Balance

R/A largely reflects the carbon balance of plants. Hence, it plays a central role in ecosystem carbon cycling and productivity assessments [13,14,15,16,20,21]. Early studies considered plant respiration to be proportional to photosynthesis, implicitly assuming a constant R/A [23,24]. Moreover, for the sake of simplicity, many vegetation models also assumed R/A (or carbon use efficiency) to be a constant [22,49]. However, accumulating evidence has indicated that R/A exhibits substantial variation across species, developmental stages, environmental conditions, and management practices [19,25,50]. Such variation may also occur among genotypes within a species. Consistent with our first hypothesis, we observed differences in R/A among the three Chinese fir genotypes (Table 1). Y020 exhibited the highest R/A (Figure 1c), indicating a substantially greater proportion of carbon consumed relative to carbon fixation than Gen3 and Y061.

Further analysis showed that the genotypic variation in R/A was primarily driven by differences in Rm (Figure 1b), whereas Am differed relatively little among genotypes (Figure 1a). This pattern was consistent with previous studies in other species indicating that respiration often drives variation in carbon use efficiency, thereby substantially influencing plant carbon balance and growth [51].

It is worth noting that no significant difference in R/A was detected between Gen3 and Y061, suggesting similar carbon balance under the conditions of this study. In contrast, Y020 had a higher R/A than Gen3 and Y061, reflecting greater respiratory carbon consumption relative to carbon fixed. The high respiration rate in Y020 might be indicative of enhanced maintenance costs or elevated investment in biosynthetic processes, such as protein turnover or cellular repair [21,26]. Conversely, the lower Rm in Gen3 and Y061 implied a potentially more conservative respiratory strategy. The observed genotypic divergence highlights the crucial role of genetic background in regulating carbon balance in Chinese fir.

### 4.2. Mechanisms Underlying Foliar Carbon Balance Variation

Numerous studies have found that NSC serve as substrates and could regulate plant respiratory metabolism [21,29]. Consistent with these findings, our results showed positive correlations between Rm and the concentrations of soluble sugars and starch (Figure 5b,e). Although previous studies have suggested that different components of NSC may have distinct effects on respiration [29,30,31], we did not observe a difference in the correlation between Rm and soluble sugars concentration and that between Rm and starch concentration. Earlier studies have shown that carbohydrate accumulation would suppress photosynthesis by feedback inhibition [52,53]. However, here, no significant correlation was found between Am and concentrations of soluble sugars and starch (Figure 5a,d). This suggests that NSC primarily affected the respiratory side of carbon balance, with limited influence on photosynthetic capacity. Partially consistent with our second hypothesis, R/A was positively correlated with soluble sugars and starch concentrations (Figure 5c,f). This suggests that NSC could serve as a key physiological factor in regulating plant carbon balance [19,21]. For the three Chinese fir genotypes, Y020 had higher soluble sugars and starch concentrations than Gen3 and Y061, consequently resulting in a greater capacity for carbon turnover and metabolic activity. Such difference in carbon reserves among genotypes may be attributable to variations in phloem loading capacity, storage tissue structure, or carbon allocation priorities [54], although the specific mechanisms remain to be elucidated.

Foliar N is one of the key factors regulating both photosynthesis and respiration [11,26,48]. However, in our study, only Rm was positively correlated with foliar N, whereas the relationship between Am and foliar N was weak (Figure 5g,h). This may be attributed to the generally high foliar N across all genotypes, which resulted in limited variation in foliar N [50]. Moreover, an earlier study showed that alterations in foliar N could affect foliar carbon balance [28]; this is likely because the proportion of nitrogen allocated to chloroplasts and mitochondria varies with nitrogen supply, thereby altering the balance between carbon fixation and consumption [28,55]. Nevertheless, we found no significant correlation between foliar N and R/A (Figure 5i), indicating that our second hypothesis was not fully supported.

### 4.3. Potential Ecological Implications of Genotypic Variation

As partially discussed above, we observed that the three studied Chinese fir genotypes profoundly differed in most examined foliar biochemical and physiological traits. Overall, PCA indicated that Y020 was primarily distributed at the positive end of PC1, Gen3 at the negative end of PC1 and the positive end of PC2, and Y061 at the negative end of both PC1 and PC2, highlighting distinct differentiation in carbon and nitrogen metabolic traits. Among the three genotypes, Gen3 had greater carbon fixation capacity (i.e., Am) than the other two genotypes. This makes Gen3 particularly suitable for the establishment of high-productivity and carbon sink-oriented plantations, especially in nutrient-rich sites where rapid growth and high carbon accumulation are desired. In contrast, Y020 showed slightly lower photosynthetic capacity than Gen3 but higher soluble sugars and starch concentrations (i.e., NSC) in the leaves. High NSC would facilitate osmotic adjustment and stress tolerance, conferring strong adaptation to abiotic stresses such as drought and high temperatures [29,54,56]. However, this strategy may come at the cost of high respiratory consumption and increased R/A (reduced carbon use efficiency), and A/N and foliar C/N (lowered nitrogen use efficiency), thereby limiting its long-term carbon sequestration potential. Therefore, Y020 is better suited for regions prone to climatic extremes, but should be used with caution in carbon sink-oriented plantations or nutrient-poor sites. While Y061 had the lowest photosynthetic rate among the genotypes, its high SLA, high A/N and foliar C/N, and low R/A collectively reflected a high efficiency in resource acquisition and utilization. This makes it potentially suitable for nutrient-poor environments, although its tolerance to abiotic stresses may be limited.

## 5. Conclusions

Our study provides novel insights into variation in foliar carbon balance among Chinese fir genotypes. Having fully supported our first hypothesis and partially supported the second, we found that foliar carbon balance (R/A) varies among genotypes and is associated with foliar NSC concentrations, including both soluble sugars and starch.

## Figures and Tables

**Table 1:** One-way ANOVA testing genotypic effects on foliar gas exchange and physiological traits.

Trait	F(2, 14)	*p* Value
Am	5.059	0.022
Rm	10.06	0.002
R/A	11.64	0.001
SLA	3.771	0.049
Starch concentration	6.083	0.013
Soluble sugars	17.21	<0.001
Foliar N	6.187	0.012
Foliar C	9.511	0.002
Foliar C/N	6.79	0.009
A/N	2.689	0.103
R/N	3.911	0.045

Abbreviations and units: Am, mass-based photosynthetic rate (nmol g^−1^ s^−1^); Rm, mass-based respiration rate (nmol g^−1^ s^−1^); R/A, the respiration-to-photosynthesis ratio (unitless); SLA, specific leaf area (cm^2^ g^−1^); Foliar N, foliar nitrogen concentration (mg g^−1^); Foliar C, foliar carbon concentration (mg g^−1^); Foliar C/N, the ratio of foliar carbon concentration to foliar nitrogen concentration (unitless); A/N, the ratio of photosynthesis to foliar nitrogen concentration (nmol CO_2_ mg^−1^ N s^−1^); R/N, the ratio of respiration to foliar nitrogen concentration (nmol CO_2_ mg^−1^ N s^−1^).

## Data Availability

The original contributions presented in this study are included in the article. Further inquiries can be directed to the corresponding authors.
